# An Adaptive Cue Selection Model of Allocentric Spatial Reorientation

**DOI:** 10.1037/xhp0000950

**Published:** 2021-10

**Authors:** James Negen, Laura-Ashleigh Bird, Marko Nardini

**Affiliations:** 1 School of Psychology, Liverpool John Moores University; 2 Institute of Cognitive Neuroscience, University College London; 3 Department of Psychology, Durham University

**Keywords:** reorientation, Bayesian development, spatial development, spatial cognition

## Abstract

After becoming disoriented, an organism must use the local environment to reorient and recover vectors to important locations. A new theory, *adaptive combination*, suggests that the information from different spatial cues is combined with Bayesian efficiency during reorientation. To test this further, we modified the standard reorientation paradigm to be more amenable to Bayesian cue combination analyses while still requiring reorientation in an allocentric (i.e., world-based, not egocentric) frame. Twelve adults and 20 children at ages 5 to 7 years old were asked to recall locations in a virtual environment after a disorientation. Results were not consistent with adaptive combination. Instead, they are consistent with the use of the most useful (nearest) single landmark in isolation. We term this *adaptive selection*. Experiment 2 suggests that adults also use the adaptive selection method when they are not disoriented but are still required to use a local allocentric frame. This suggests that the process of recalling a location in the allocentric frame is typically guided by the single most useful landmark rather than a Bayesian combination of landmarks. These results illustrate that there can be important limits to Bayesian theories of the cognition, particularly for complex tasks such as allocentric recall.

## Public Significance Statement

Whether studying the development of children’s spatial cognition, creating artificial intelligence with human-like capacities, or designing civic spaces, we can benefit from a strong understanding of how humans process the space around them. Here we tested a prominent theory that brings together statistical theory and psychological theory (Bayesian models of perception and memory) but found that it could not satisfactorily explain our data. Our findings suggest that when tracking the spatial relations between objects from different viewpoints, rather than efficiently combining all the available landmarks, people often fall back to the much simpler method of tracking the spatial relation to the nearest landmark.

Reorientation is the process of recovering one’s heading and position in a given space. This is a process that allows a disoriented organism to recover the correct vector to important locations. The ability to do this is a key adaptation for the vast majority of mobile organisms. The study of how humans and other animals do this has moved forward our understanding of both cognition (Lee, 2017; Mou & Zhou, 2013; Nardini et al., 2009; Twyman et al., 2018) and the mammalian brain (Cressant et al., 1997; Ito et al., 2015; Keinath et al., 2017; Park et al., 2011). This has especially become a crucial point in developmental studies of spatial cognition, igniting a debate over modular cognition (Cheng, 1986; Doeller & Burgess, 2008; Hermer & Spelke, 1996, 1994) versus adaptive behavior (Cheng et al., 2013; Learmonth et al., 2002; Ratliff & Newcombe, 2008b; Twyman et al., 2018). A recent article formalizes and details a specific proposal concerning adaptive behavior (Xu et al., 2017). More than just adaptive, this new theory posits that children’s use of different cues to reorient is fully rational and Bayesian. The full name of the model is the adaptive cue combination model of human spatial reorientation. For brevity, we will refer to it as *adaptive combination*. The present study seeks to further test this model as a general way of understanding how humans, especially young children, reorient themselves to find goal locations.

Adaptive combination is an important model for the study of developing spatial cognition. Despite decades of research (Cheng et al., 2013; Lee, 2017; Miller, 2009), there are still debates about the way that different cues are used by young children to reorient. For example, an early theory posited that reorientation only depends on environmental surfaces or boundaries, with the exception of adults who have a linguistic mechanism of incorporating additional information (Hermer & Spelke, 1994). In other words, if the target was to the right of a wall that was relatively short and coloured blue, an adult can synthesize the two pieces of information (right of short + right of blue) into one linguistic construct that could guide behavior: “to the right of the short blue wall.” This theory, like many after it, faced a serious difficulty. It was discovered that young children’s performance can be improved through the addition of a nonboundary cue as long as the room is sufficiently large (Learmonth et al., 2002). This showed that the process is not purely dependent on boundary information, even in young children. The present article seeks to test adaptive combination independently in the hopes of leading toward a consensus on how developing spatial cognition handles multiple reorientation cues.

If adaptive combination is true, it is also a breakthrough finding for the study of developing Bayes-like reasoning in perception and memory. Almost all previous studies to look at Bayesian cue combination in children under 10 years old have returned negative results (Adams, 2016; Burr & Gori, 2011; Chambers et al., 2018; Dekker et al., 2015; Gori et al., 2012; Jovanovic & Drewing, 2014; Nardini et al., 2010, 2013; Petrini et al., 2014), including one that looked at combination of cues for spatial recall (Nardini et al., 2008). For example, when judging a horizontal location with a spatialized audio cue and a brief visual cue, children under 10 years old fail to integrate the two efficiently; the precision of their judgements is not any better than with the visual cue alone (Gori et al., 2012). If the process of reorientation really does happen with full Bayesian efficiency, this means that spatial cognition is an exception to the general rule. Children might begin reasoning in a Bayes-like way in terms of reorienting first, then extend this to other cognitive processes throughout childhood. This again makes it vital to see if this theory can be verified independently: It has serious potential impact in terms of both spatial cognition and a general theory of how Bayesian reasoning develops.

The remaining sections of the introduction (a) detail this model and define further terminology, (b) specify the gaps in evidence for adaptive combination, (c) explain key choices in the present study’s design, and (d) detail specific hypotheses and the way they will be tested.

## The Adaptive Combination Model and Terminology

First, we need to make it clear what the adaptive combination model is and how it works. The article grounds the model first in optimal Bayesian principles but largely leaves aside the issues of prior distributions (assumed to be uniform for all the data they model) and the question of explicit decision rules. Rather, they insert a number of typical assumptions into the broader Bayesian framework until the model is governed by a central law which specifies the way that multiple cues are used. That law is given in the article in relation to four specific cues, reflecting the data they had available. That law can be stated generally as

$$\;f_{1+2}\left(\text{Response}|\text{Target}\right)\propto f_{1}\left(\text{Response}|\text{Target}\right)\times f_{2}\left(\text{Response}|\text{Target}\right),$$ 1

where the function $f_{1}\left(\text{Response}|\text{Target}\right)$ specifies the probability of given responses to given targets with only the first cue, $f_{2}\left(\text{Response}|\text{Target}\right)$ does the same for the trials with only the second cue, and$\;f_{1+2}\left(\text{Response}|\text{Target}\right)$ governs responses when both cues are presented at the same time. One can view this as the core pattern of interest when applying Bayesian cue combination models to cognition: under typical assumptions, it (a) respects Bayesian principles, (b) optimally integrates the information given by both cues, and (c) minimizes uncertainty (Rohde et al., 2016). We discuss this with the term *Bayesian cue combination*, but the reader should also be aware that Bayesian principles can result in additional and/or different predictions if other assumptions are inserted—for example, if a nonsymmetric loss function is used. The reader should also be aware that the same pattern can be motivated through a maximum likelihood framework (Ernst & Banks, 2002).

**Figure 1 fig1:**
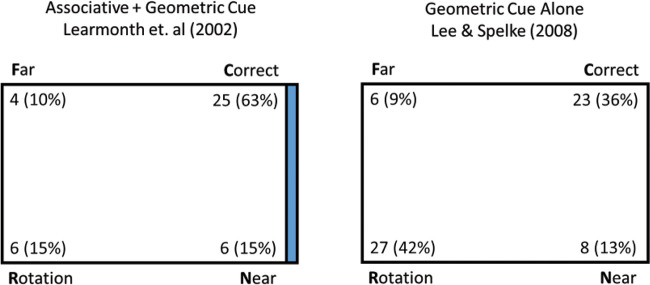
Examples of Previous Results With Reorientation Tasks *Note*. Children were placed in a rectangle arena with four hiding locations, one in each corner. The target was hidden in the corner marked “correct.” Children were first disoriented and then allowed to search for the target. On the right, participants can only use the geometry to find the target. This means that they respond in roughly equal numbers at the correct corner and its rotational equivalent (i.e., both corners with a long wall to the left and a short wall to the right). On the left, one of the walls was colored blue, while the others were white. This associative cue made it possible to disambiguate the correct corner and its rotational equivalent. Children responded more often at the correct corner. See the online article for the color version of this figure.

To further understand Equation 1, an example might be helpful. Some more terminology will be needed. In the typical reorientation paradigm (see Figure 1), children are placed in a rectangular arena and shown a target hidden in one corner. They are disoriented and then released to search one of the corners for the target. The correct corner is conventionally called *C* (for correct), the rotational equivalent called *R* (for rotational equivalent), the corner on the same short wall as the target called *N* (for near), and the corner on the same long wall called *F* (for far). If the geometry of the room is the only available cue, this is a *G* condition (for geometry). If there is also something unique about one of the walls to associate with the target, then it is an A + G condition (i.e., Associative + Geometry).[Fig-anchor fig1]

We can now insert some specific numbers and give example calculations. With boundary geometry alone, suppose participants respond at C 40% of the time, R 40%, N 10%, and F 10%. That is $\;f_{1}\left(\text{Response}|\text{Target}\right)$. Suppose that an associative cue alone would point a child to C 40%, R 10%, N 40%, and F 10%. That is $\;f_{2}\left(\text{Response}|\text{Target}\right)$. Assuming that adaptive combination is correct, we can now predict how often they will respond at each location during an A + G condition. We multiply to obtain P(C) = .4 × .4 = .16, P(R) = .04, P(N) = .04, and P(F) = .01. These then must be normalized (divided by their sum) to arrive at the final probabilities. Those are P(C) = 64%, P(R) = 16%, P(N) = 16%, and P(F) = 4%. That is $\;f_{1+2}\left(\text{Response}|\text{Target}\right)$.

Equation 1 can lead to a variety of different interesting patterns, but one will be particularly critical here. In the example, the two cues presented together led to a higher proportion of correct answers (64%) than either cue alone (40%). In general, if both f_1_ and f_2_ have some concentration (a discrete mode or a continuous peak) in the same place, then f_1+2_ will have an even greater concentration around the same place. In the case in which *f*_1_ and *f*_2_ are normal distributions, *f*_1+2_ will be a normal distribution with precision (1/variance) that is additive: it will be equal to the sum of the precisions of *f*_1_ and *f*_2_.

The general framework for Bayesian cue combination, crucially for our purposes here, is completely agnostic about any detail beyond Equation 1. It has no preference or disregard for any kind of cue. It does not matter what domain the task is within (e.g., spatial memory, speech perception, weight perception, etc.). It works the same way if the two cues are very different, such as a boundary and a local landmark, or if they are very similar, such as two landmarks, or even if they are the exact same stimulus played repeatedly (Jones, 2018). It functions for either continuous responses or discrete nominal responses. It only matters that *f*_1_, *f*_2_, and *f*_1+2_ can be specified.

## The Need for Additional Scrutiny

Second, we need to clarify where the gaps in evidence for adaptive combination exist. In the literature on Bayesian perception and decision making, there is a standard set of three findings that are used to show that two cues are combined in a Bayesian manner. This is routine enough that it has been codified into a tutorial with supporting R packages for the case of normal distributions (Rohde et al., 2016). The procedure measures how precise participants are with one cue in isolation, the other cue in isolation, and both cues together. It then must be shown that (a) precision is better with both cues versus the first cue in isolation, (b) precision is better with both cues versus the second cue in isolation, and (c) precision is not significantly different with both cues versus the Bayesian optimal prediction (predicated on Equation 1). These findings rule out the alternative hypothesis that either single cue is being used in isolation; otherwise, we would not expect better precision when both are presented. These findings also speak against the alternative hypothesis that the two cues are being used together in some non-Bayesian fashion; since Bayesian cue combination is the optimal way to improve precision, no other process could also match the optimal Bayesian precision.

Although the procedure above is designed for assessing cue combination in the case of normal distributions, it adapts readily to discrete nominal distributions. It should still hold that (a) there is a difference between the response distribution with one cue versus both cues, (b) there is a difference between the response distribution with the other cue versus both cues, and (c) the distribution of responses with both cues is not significantly different than the prediction given by Equation 1. This set of findings rule out the possibility that any single cue is being used in isolation. They also speak against the alternative hypothesis that the two are being used in some way that does not conform to Bayesian cue combination.

Unfortunately, the article arguing for adaptive combination (Xu et al., 2017) provides only one of the three pieces of evidence. Specifically, it reviews evidence that performance with A + G conditions differs from performance with G conditions. It does not show that performance with A + G conditions differs from performance with A conditions (where only an associative cue is presented; in practice, a square room with a single uniquely colored wall). It also does not use data from G conditions and A conditions to derive predictions for A + G conditions and compare that to A + G data. This leaves open the alternative hypothesis that children may complete an A + G condition by only using the associative cue. Twyman and colleagues (2018) also pointed out the need for this type of data in their discussion (p. 934).

We tried to fill this gap as best as possible by looking through the available literature. Unfortunately, this attempt failed to show that performance in A + G conditions is different than performance in A conditions. We reexamined previous data for an A + G condition (Learmonth et al., 2002) and an A condition (Hermer-Vazquez et al., 2001). Since results are known to depend on age, we used the data from 5 year olds from both studies. As Adaptive Combination is theorized to ignore associative cues in small rooms, rather than combine them, we also used the data from the larger room in the Learmonth et al. (2002) study. Analysis suggests that the two distributions are not reliably different, χ^2^(3) = 2.08, *p* = .56. Further, a Bayesian version of this analysis can test the hypothesis that the response distributions are the same versus the hypothesis that they are different. This analysis results in BF_01_ = 18.44, considered “strong” evidence that they are the same. We would present additional analyses, but A conditions are relatively rare in the literature, and this was the only comparison we could find with a sufficient age match, the standard methods described earlier, and a full report of the response distributions.

There is also another project that examined cue combination in reorientation (Wang et al., 2018), but it also leaves further need for investigation. They used streets (S) and buildings (B) as cues in an adult sample. On the one hand, there was no significant difference between dual-cue SB performance and the predictions of their combination model. Further, a BIC analysis favored a combination model over a single-cue model. On the other hand, there was no significant difference between single-cue B performance and dual-cue SB performance in either experiment. In addition, the BIC delta was 4.0—a result that is typically considered “positive” but not “strong” or “decisive” (Kass & Raftery, 1995). Further, since it was an adult sample, it does not particularly help resolve questions about development. It is ultimately an interesting but mixed set of results that leave open the need for further study.

From the point of view of the literature on Bayesian perception and decision making, this makes it clear that further evidence is needed for the adaptive combination model. The reanalysis of the available previous data suggests that Bayesian reasoning is not occurring here. Instead, it suggests that participants in an A + G condition are merely using the associative cue to complete the task. This is certainly an unusual interpretation—to our knowledge, it has not previously been tested if performance in an A + G condition might depend entirely on the use of the associative cue. However, it may still be possible to improve on this analysis. This will be described in more detail in the next section, but briefly: The number of trials per participant is (radically) smaller than most cue combination studies, it is not ideal to use cues that are not equally useful, and it is not ideal to use between-subjects data. We therefore designed a new study to test adaptive combination in a more rigorous fashion.

## The Present Study

Third, we now outline key design decisions for the study. To do this, we need to comment on our focus with this design. We need to draw a distinction between *reorientation*, the process of regaining a sense of place and heading to find goal locations, and *the reorientation paradigm*, a common method where participants are placed in a rectangular room and asked to find a target in one of the corners. We are interested in reorientation. We are not directly interested in the reorientation paradigm itself; we are only interested in it to the extent that it provides information about reorientation. At first, this may seem to put us at odds with the authors of the adaptive combination model because they used only data from the reorientation paradigm. However, this is not the case. A full and careful reading of their article indicates that they are not aiming only to understand the particulars of how young children deal with being turned around in a rectangular room with a blue wall. It makes sense that they modeled the classic reorientation paradigm because those were the data that were available in great enough quantities to model in a meaningful way with their approach. However, the goal of the article, like ours, is to examine a general model and principle that could be a unified explanation for behavior across different environments and across development. From our point of view, it will be a major strength if adaptive combination can predict outcomes in a reorientation task that falls outside the reorientation paradigm; if it cannot, we view this as a limitation that is at least worth considering. Based on our overall view, we chose to fashion our task toward the best test of the underlying Bayesian mechanics without regard to the typical reorientation paradigm.

Any cue combination study must overcome several routine problems (Rohde et al., 2016), all of which make a standard A + G method less than ideal. First, the two cues to be presented should ideally be matched in their reliability; participants should be about as precise with either cue. This is the situation in which the potential benefit of combination is greatest, and so the one in which the Bayesian optimal prediction is as different as possible from the alternative hypothesis that only one cue is being used. Second, it is also ideal to use a task for which the noise in perception/memory is approximately normally distributed around the target. This makes it possible to analyze precision (1/variance), which generally provides more statistical power than discrete nominal distributions. It also makes it possible to use simple, standard ways of predicting the optimal precision (Ernst & Banks, 2002). Third, it is ideal to use a situation where each participant can provide the highest possible number of trials, allowing for a within-subjects design. This makes it possible to calculate individual predictions for Bayesian optimal precision and compare these to individual measurements of precision with both cues. None of these three conditions are met in a standard A + G condition: the associative cue is more reliable than the geometric cue (Hermer-Vazquez et al., 2001; Lee et al., 2012; Nardini et al., 2009), the errors are discretely distributed, and young participants will not generally tolerate much more than four trials in total.

Instead, we adapt a method from previous studies (Negen, Heywood-Everett, et al., 2018; Negen, Roome, et al., 2018). Virtual reality is used to make the trials faster and to make the task more engaging. Participants are shown a target being hidden among some landmarks. They then have their view blocked while their perspective changes. From this new perspective, the participant attempts to point where the target was hidden. On some trials, there is a pair of landmarks marking the North South (N/S) axis of the space, called NS trials. On other trials, there is a pair of landmarks marking the East West (E/W) axis, called EW trials. In the last kind of trial, both pairs of landmarks are available. We refer to this as an NSEW trial. (Throughout the article, we use NS or EW to mean a trial type and N/S or E/W to mean an axis of the space.) This allows us to measure performance with two different cues (landmark pairs) in isolation and with both together. Participants included both adults and children (5 to 7 years) because adaptive combination is supposed to apply across the life span.

This design overcomes the usual problems described above. Because both cues are landmark pairs, they are matched in reliability. Responses on this kind of task are approximately normally distributed around the targets. Because more trials are tolerated, a within-subjects design is possible. This makes it a good way to test if reorientation cues are used together in a Bayesian manner. In that sense, the present study is a test of a general version of adaptive combination rather than a test of its ability to explain the specific reorientation paradigm that is so prevalent in the literature.

## Hypotheses

**Figure 2 fig2:**
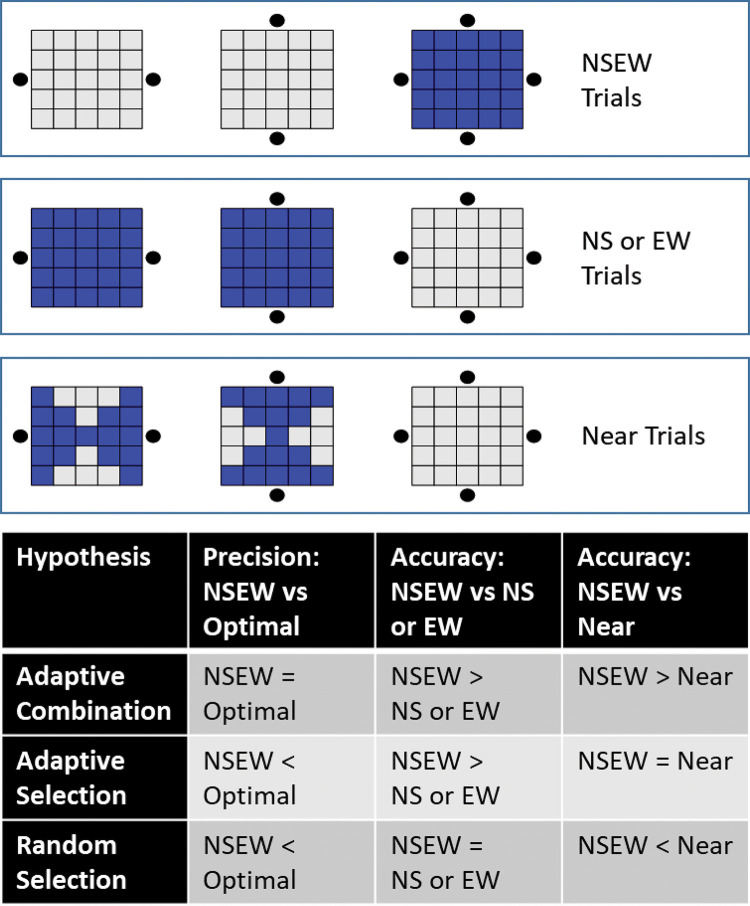
Trial Types and Key Predictions *Note*. In terms of the methods, every trial is a NS trial (with North [N] and South [S] landmarks visible), an EW trial (East [E] and West [W] visible), or a NSEW trial (all four visible). However, for the analysis to differentiate between hypotheses, it is useful to regroup the trials. On the top half of this figure, the blue squares indicate which trials are included in each of the three regrouped categories. There are 25 possible targets in a 5 × 5 grid. The black dots indicate which landmarks are visible during those trials. NSEW includes all trials where all four landmarks were visible. NS or EW includes all trials where only the North and South landmarks were visible, plus the trials where only the East and West landmarks were visible. Near trials are a subset of NS or EW trials where the participant has the nearest possible landmark (or at least one of them if several are equidistant). The bottom tabular display gives predictions and shows that these regroupings allow us to test different predictions from the different hypotheses. See the online article for the color version of this figure.

Fourth (and finally), we detail the specific hypotheses and what predictions they make. For each of the three hypotheses, we first give a conceptual description in the top paragraph, followed by a bottom paragraph that lays out and justifies the specific empirical predictions about three outcome measures. Figure 2 is a reference guide for the different trial types and the empirical predictions of each hypothesis. The Appendix contains simulations that verify these are the correct predictions.[Fig-anchor fig2]

## Main Hypothesis: Adaptive Combination

As governed by Equation 1, the participant combines the information from the two landmark pairs in the optimal Bayesian fashion. This is a new extension (Xu et al., 2017) of the adaptive behavior proposal (Cheng et al., 2013), suggesting that participants are not only taking account of which cues are available and which one is best, but also combining different cues while weighting them in line with Bayesian principles. This would be in line with how adults perform in many simple perceptual tasks (for review, see Pouget et al., 2013). If this hypothesis fits children’s performance, that would break with the general pattern of children under 10 failing to show Bayesian reasoning (Burr & Gori, 2011) and warrant the exploration of a new theory of how Bayesian reasoning develops.

Figure 2 defines which trials are considered NSEW trials, NS or EW trials, and near trials. The adaptive combination hypothesis predicts that precision in NSEW trials will be equal to the optimal Bayesian precision. In other words, the optimal Bayesian process should produce the optimal Bayesian precision. There is a simple and well-known formula used to predict the optimal Bayesian precision (Ernst & Banks, 2002). This hypothesis also predicts that NSEW accuracy will be better than NS or EW accuracy. This is predicted because the Bayesian process should always benefit from additional landmarks—NSEW trials have four landmarks, but NS or EW trials have only two. NSEW accuracy should also be better than Near accuracy for the same reason (near trials also have only two landmarks).

## Alternative Hypothesis: Adaptive Selection

If participants do not use multiple cues with full Bayesian efficiency, they may still adopt a sensible strategy that constrains error while only using one landmark. Under adaptive selection, participants select the landmark nearest to the target, encode the target location against it, and ignore the other landmarks. In doing so, they improve average performance over just using a random landmark—but not as consistently as a Bayesian process would. This is more in line with older forms of the adaptive proposal (Cheng et al., 2013). It posits that children take account of which cue will be most useful and use this to guide which cue they use, but does not entail any combination of landmarks, that is, Bayesian reasoning. This would be in line with previous results where children are able to select the best single cues. For example, they tend to prefer visual cues for judging spatial relationships and auditory cues for judging temporal relationships (Gori et al., 2012). However, it would not allow for any new conclusions regarding Bayesian reasoning in development.

This hypothesis predicts that precision in NSEW trials will be worse than the optimal Bayesian prediction. In other words, a nonoptimal non-Bayesian process should not lead to the optimal Bayesian precision. This hypothesis also predicts that accuracy in NSEW trials will be better than accuracy in NS or EW trials overall, because the NSEW trials will sometimes have a nearer (better) single landmark to select and use. For example, look at the top middle target in Figure 2. The nearest landmark, at the North, is visible on every NSEW trial. However, it is not present during half of the NS or EW trials. This should drive higher NSEW accuracy than NS or EW accuracy. However, near accuracy should be equivalent to NSEW accuracy because they both provide the nearest (best) possible landmark to select and use. For example, looking again at the top middle target, the North landmark is visible for all NSEW trials and all near trials.

## Null Hypothesis: Random Cue Selection

On a trial with both landmark pairs, the participant chooses one landmark at random and encodes the target against it. The other landmarks are ignored. In essence, under this hypothesis, an NS or EW trial is an NSEW trial where we have done some of the random choosing for the participant. This would be similar to how children performed in a previous spatial task with two cues available, alternating in an unpredictable way between self-motion information and landmark information (Nardini et al., 2008). However, again, it would not allow for any new conclusions regarding early Bayesian reasoning.

This predicts that precision in NSEW trials will be worse than the optimal prediction. This is again because the nonoptimal non-Bayesian process should not produce the optimal Bayesian precision. It also predicts that accuracy in NSEW trials will not be different from accuracy in NS or EW trials overall. For example, we can look at the top middle target in Figure 2 again. On a NSEW trial, we only expect them to use the North (best) landmark on one out of four trials. We would expect the same thing for NS or EW trials (two trials would have the North and South available, with the North selected on one trial). This hypothesis further predicts near accuracy will be better than NSEW accuracy. For near trials, we would expect them to use the North landmark two times out of four. In other words, in a Near trial, the lack of poor encoding choices should actually help participants if they are choosing encoding references randomly.

## Experiment 1

### Method

Ethics approval was granted by the Durham University Psychology Ethics Committee (Reference: 09/15 Development of Spatial Cognition).

#### Participants

There was total of 36 participants tested. Of these, 12 were adults (seven women). They ranged from 18 to 23 years old (*M* = 20.9 years, *SD* = 1.25 years). The remaining 24 were children. Four did not complete the task, one because the headset was too large and three due to mood. Of the remaining 20 (four girls), they ranged in age from 5 years and 0 months to 7 years and 5 months, with a mean of 6.1 years and a standard deviation of .6 years. All participants were recruited in the Northeast U.K. area. To the knowledge of the researchers, no children had been diagnosed with any perceptual or developmental disorder that might have affected task performance. The advertisements asked only for participants with normal vision or vision that could be corrected to normal with contact lenses. Adult participants (Psychology undergraduates) earned credits in a scheme where undergraduates participate in each other’s research projects. Children were given a small toy of their choosing. Written informed consent was obtained, either from the adults themselves or the parents of the children. Verbal assent was also obtained from the children themselves. Given the three specific hypotheses and the large effect sizes expected between them, we were comfortable with the power given by 12 adults or 20 children: 80% power at an effect size of .76 or .58.

#### Apparatus

**Figure 3 fig3:**
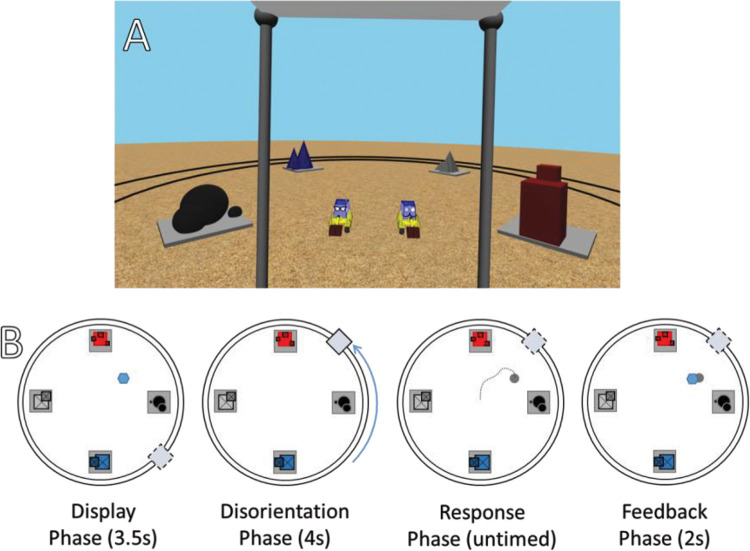
Methods for Experiment 1 *Note*. (A) A first-person screenshot of the view within the experiment. Please be aware that the lenses of the oculus rift slightly distort the internal screen image, so the image given to it is distorted in the opposite way. For example, in the headset, the red and blue landmark clearly face each other directly; in the screenshot, they appear slightly offset. (B) First, the target (blue hexagon) was shown to the participant while they were in the cart (dashed box). Then the cart “closed,” blocking their view, and the participant was moved +90, –90, or 180 degrees around the track (black circles). Then the view was opened, and the participant moved a gray cone to the point where they believed the target to be. Finally, feedback was given as to the correct placement. This could be done with the North and South landmark (red and blue), the East and West landmark (gray and black), or all four. See the online article for the color version of this figure.

The study used Vizard 5 (Vizard, 2018) and the Oculus Rift headset (Consumer Version, Oculus, Menolo Park, CA). It also used an Xbox One controller (Microsoft, Redmond, WA). The virtual world (see Figure 3) contained three major features situated around a 5-m × 5-m virtual space. First, there was a set of train tracks in a circle around the central space with a small cart. The cart could move around the tracks and had opaque shutters that could come up and down. The participant’s perspective was always from within the cart. Second, there was a set of four landmarks which could fade in and out of view. They were each unique and distinctive: black spheres, gray pyramids, red blocks, and blue cones. With the center of the space at (0, 0), these were placed at the four cardinal points: (0, 3) (0, –3) (3, 0), and (–3, 0) in meters. Third, there were the diggers. These were the characters that played the game with participants. To make them more engaging to the children, they were given silly names and apparel. One digger, who had a moustache and wore a pipe hat, was named Digger T. Diggington, III. The other digger, who wore a set of glasses with jewels and a large feather attached to a band around her head, was named Martha Diggington, Esquire. The 3D models for the Diggingtons had joints in the digging arm to their front so that they could be animated as digging a place for the target and then digging it back up. Fourth, there were the jewels. These served as targets to find. They were translucent blue (80% opacity) and fashioned after a round brilliant cut. There were no other landmarks or features in the environment that could be used to reorient (e.g., the skybox was uniform blue). The ground had a repeating sand texture at 20% opacity.[Fig-anchor fig3]

#### Procedure

The game began by allowing the participant to select the character they wanted to play with. The other character faded out of view. The first warmup trial began.

Each trial involved a series of four steps (see Figure 3). First, the target was shown. The digger went to the target location. They stayed there for 3.5 s while an animation played of the target (jewel) being buried. The last .5 s involved the jewel going 3 m into the air and moving straight down into the ground to make it as clear as possible exactly where it was.

Second, there was the disorientation. The opaque shutters on the cart moved up to block the participant’s vie, and 3 s elapsed while a sound effect of a train moving played. The viewpoint changed. The shutters then lowered. This took a total of 4 s. Participants were told that the cart moves around the track to a new location. This disorientation procedure has the key effect of placing participants at a new, unpredictable viewpoint, although without physically turning them as has been common in some other studies.^1^

Third, there was the response. The participant used the joystick on the controller to move a large arrow with its tip on the ground within the 5-m × 5-m central space. There was a gray circle on the tip of the arrow with a radius of 75 cm. When satisfied with the location, the participant pressed a button on the controller to enter their response. They were allowed as much time as they wanted, but younger participants were encouraged to take their best guess if they said that they did not know the right place.

Fourth, there was feedback. The digger moved over to the response location and played a 2-s digging animation. If the response was within 75 cm of the target, the jewel appeared out of the ground, a small *ding* sound played, and the digger jumped up and down in a celebratory animation. If not, no jewel appeared, no sound played, and the digger turned toward the participant to play a “deflated” animation. Over the course of 1 s, their body widened along the ground plane by 20% while their height shrunk 20%. It then returned to normal shape over the next 1 s. During this, a small blue circle flashed on the ground at the correct target location. When a button on the controller was pressed again, the next trial began.

The first five trials were considered warmup trials. These data are not analyzed in any part of the results. During this time, the experimenters gave the children hints and explanations about the game. For remaining trials, participants were not given any extra information about the target location.

#### Stimuli and Trial Parameters

Target locations were on a 5 × 5 grid with 1 m spacing. For example, there was a corner target at (2, 2), a center target at (0, 0), an off-center target at (0, –1), and a target in front of the West landmark at (–2, 0). For the five warmup trials, the targets were always (0, 0) (0, 2) (2, 0) (–2, 0), and (0, –2). After that, for adults, all 25 possible target locations were used. For children, to make the game shorter, only nine target locations were used: (0, 2) (–1, 1) (2, 1) (–1, 0) (0, 0) (1, 0) (–2, –1) (1, –1) and (0, –2). These were selected to represent a range of different distances from the different landmarks and the center. Each target was tested once with the East and West landmarks (EW trial), once with the North and South landmarks (NS trial), and once with all four (NSEW trial). The order of trials was random. This means, in total, that adults produced 75 analyzed trials each and children produced 27.

The cart could travel either +90, –90, or 180 degrees around the track. This was done because the corners provided a good view of the target space where all four landmarks were visible but not obstructing the 5-m × 5-m response area. The amount of travel was chosen randomly on each trial. Each trial began wherever the last one ended.

#### Analysis Plan

To analyze these data, we planned to have four tests. First, just to confirm that the task was understood by participants, we checked that target locations and response locations were significantly correlated along both the *x*-axis and the *y*-axis. After this, responses were excluded as outliers if they were more than 2.5 standard deviations in error away from the target.

To make the next three tests clear, we need to comment on accuracy, mean error, precision, and variable error. Some of the hypotheses are stated in terms of accuracy. To be more specific, we intend this as the mean error: the average distance between the target location and the response location, calculated along the two-dimensional (2D) plane using the Pythagorean theorem. Lower mean error indicates better accuracy. The other hypotheses are stated in terms of precision. To look at precision, we analyze variable error: the standard deviation of the response locations minus the target locations (retaining the sign). As the variable error (standard deviation) of responses increases, precision decreases. Precision is conventionally defined as variable error raised to the power of negative two. Using variable error in the analyses, rather than precision, is standard practice in the cue combination literature (Rohde et al., 2016). This is because variable error tends to better approximate a normal distribution and tends to have a (much) less serious problem with sensitivity to outliers. In line with this, we analyzed variable error. Lower variable error indicates better precision. Conveniently, this means that a shorter bar denotes better performance in all of the bar graphs that will be shown. Since responses were along a 2D ground plane, there is a separate variable error along each axis of the space. We used variable error to test specific predictions about reaching the optimal Bayesian precision; otherwise, we used accuracy as a measure of performance.

For the second test, we looked at the Bayesian optimal variable error in NSEW trials, where all four landmarks were visible, versus observed variable error in NSEW trials. Adaptive combination predicts that these will be equal. In other words, an optimal Bayesian process should produce the optimal Bayesian variable error. Adaptive Selection and Random Selection suggest that observed variable error with both cues should be worse than the optimal prediction. In other words, a nonoptimal non-Bayesian process should fail to produce the optimal Bayesian variable error. For each participant, along each axis, for each trial type (NS, EW, and NSEW), we calculated the variable error. For each participant, the optimal variable error is calculated with the equation (Ernst & Banks, 2002):

$${\text{σ}}_{opt}=\;{\left({\text{σ}}_{EW}^{-2}+{\text{σ}}_{NS}^{-2}\right)}^{-\frac{1}{2}}.$$ 2

This comparison, as well as the next two, are tested with paired *t* tests. This second test conforms entirely with the standard method of testing for optimal Bayesian cue combination (Rohde et al., 2016). Because the hypotheses for this test are directional, a one-tailed test was used.

Third, we tested the accuracy in NSEW trials versus the accuracy in NS or EW trials, where only two landmarks were visible. Random Selection predicts that accuracy should be the same in NSEW trials versus NS or EW trials—under Random Selection, a NS or EW trial is just a NSEW trial where we have done some of the random selection for the participant. Adaptive selection and adaptive combination predict that NSEW accuracy should be better than NS or EW accuracy, using the additional information in a NSEW trial to improve accuracy through either selecting the best single landmark (selection) or via Bayesian cue combination (combination).

Fourth, we tested NSEW accuracy against near accuracy. A trial is considered a near trial if it is a NS trial or EW trial where a nearby single landmark is visible—at least as near as the nearest one in a NSEW trial with the same target (see Figure 2). This analysis proceeds on the assumption that accuracy at localizing a target location using a landmark decreases as the target location gets further from the landmark (e.g., Negen, Roome, et al., 2018). Random selection predicts that NSEW accuracy should be worse than near accuracy, since the participant will sometimes randomly select one of the landmarks from the further (worse) pair to use on a NSEW trial. Adaptive selection predicts that NSEW and near accuracy should be equal because participants complete a NSEW trial by only using the nearest landmark anyway. Adaptive combination predicts that NSEW accuracy should be better than near accuracy because the Bayesian framework allows information from the further (worse) landmarks to be incorporated in a way that it still improves the responses.

Bayes factors (BFs) for *t* tests and correlations were calculated using an online tool (Rouder et al., 2009) and BFs for analyses of variance (ANOVAs) were calculated with Jamovi 1.8.1 (Jamovi, 2021). The notation BF_10_ indicates support for the alternative hypothesis and the notation BF_01_ indicates support for the null hypothesis.

### Results

**Figure 4 fig4:**
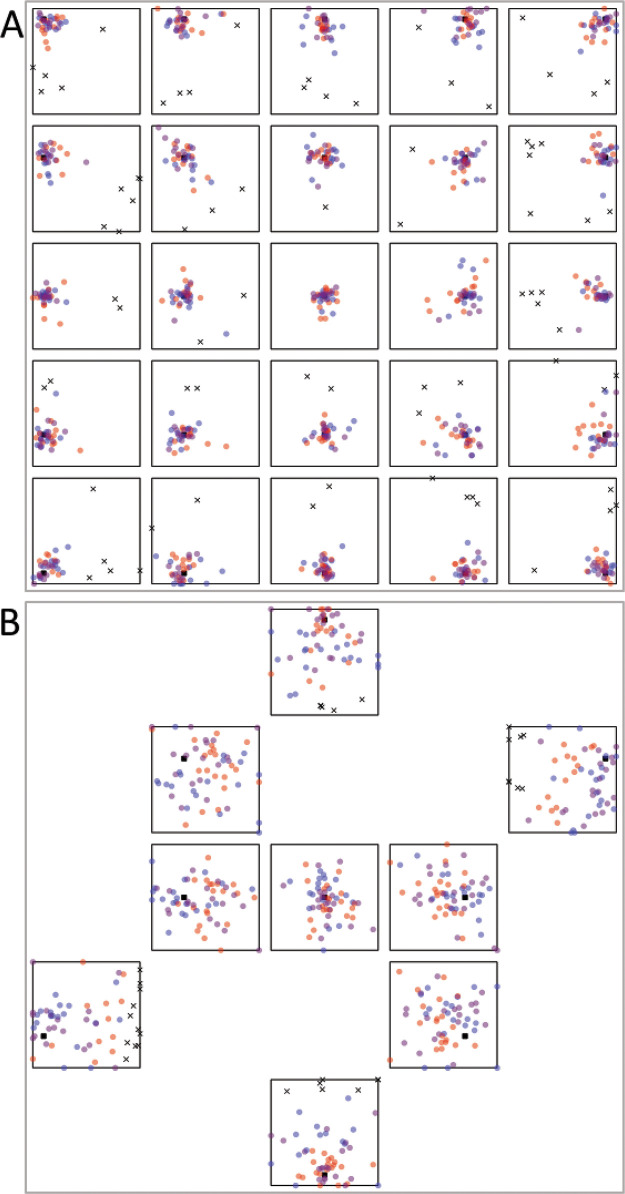
Adult (A) and Child (B) Data From Experiment 1 *Note*. Red dots are responses on NS trials, where the North (N) and South (S) landmark are visible. Blue dots are East West (EW) trials. Purple dots are NSEW trials. The black square is the target. Black crosses are excluded trials. See the online article for the color version of this figure.

**Figure 5 fig5:**
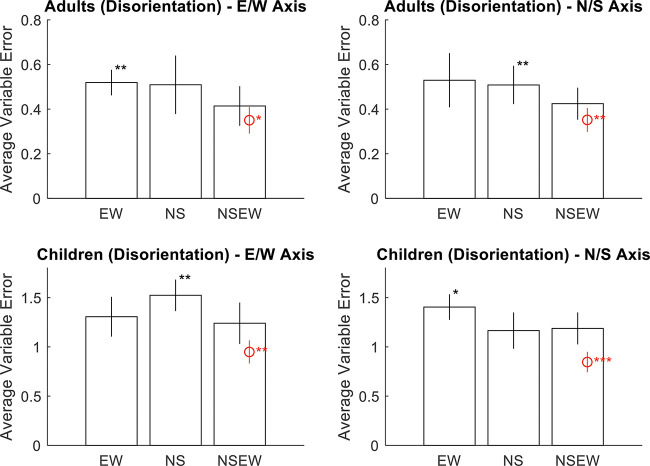
Average Variable Error Broken Down by Trial Type (x-Axis), Participant Group (Top Versus Bottom Panels), and Axis of the Space (Left Versus Right Panels) *Note*. Error bars are 95% confidence intervals for the mean. Asterisks mark significant paired *t* tests against NSEW. The red marking is the optimal prediction. Along both axes, participants had significantly higher variable error than the optimal prediction when shown all landmarks. This speaks against adaptive combination but is consistent with either adaptive selection or random selection. NSEW = North South East West. * *p* < .05. ** *p* < .01. *** *p* < .001. See the online article for the color version of this figure.

**Figure 6 fig6:**
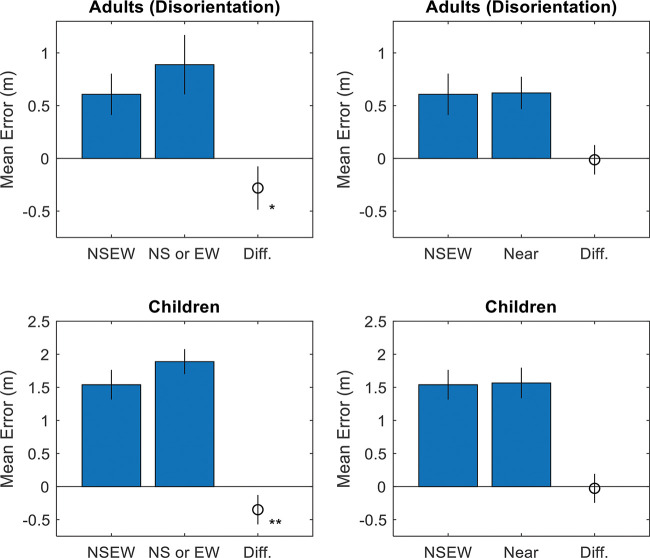
Accuracy and NSEW Trials *Note*. NSEW trials are broken down by group (top vs. bottom panels), trial type (*x*-axis), and comparison trials (left versus right panels). Results favor adaptive selection, which predicts a difference versus NS or EW trials but not versus near trials. NSEW = North South East West. * *p* < .05. ** *p* < .01. See the online article for the color version of this figure.

Results strongly favor adaptive selection for both adults and children. See online supplemental materials for raw data. For adults, the responses were correlated with the targets along the *x*-axis, *r*(898) = .83, *p* < .001, BF_10_ = 3.52 × 10^225^, and the *y*-axis, *r*(898) = .80, *p* < .001, BF_10_ = 1.37 × 10^197^ (see Figure 4). Responses were excluded if they were more than 2.5 standard deviations away from the target (2.1m; 4.3% or 77 observations). Variable error was worse (higher) than the Bayesian optimal variable error along both the E/W axis, *t*(11) = –1.97, *p* = .038, *d* = –.57, BF_10_ = 1.72, and the N/S axis, *t*(11) = –2.76, *p* = .009, *d* = –.80, BF_10_ = 5.04 (see Figure 5). Accuracy was better in NSEW trials versus the NS or EW trials, *t*(11) = –3.02, *p* = .012, *d* = –.87, BF_10_ = 7.23 (see Figure 6). Accuracy was not better in the NSEW trials versus the near trials, *t*(11) = .21, *p* = .839, *d* = .06, BF_01_ = 2.59.[Fig-anchor fig4][Fig-anchor fig5][Fig-anchor fig6]

If adaptive combination were correct, we would not expect to see a difference between the optimal variable error and the observed variable error with both cues. We would also expect to see that NSEW accuracy was better than near accuracy. If random selection were correct, we would not expect to see a difference between NSEW accuracy versus NS or EW accuracy. We would also expect to see that NSEW accuracy was worse than near accuracy. In other words, both adaptive combination and random selection were positively ruled out by statistically significant findings. In contrast, adaptive selection correctly predicted that the variable error with both cues would be worse than optimal, that NSEW accuracy would be better than NS or EW accuracy, and that there would not be a difference between NSEW accuracy and near accuracy.

For children, the pattern was the same (but with worse variable error and accuracy). The responses were correlated with the targets along the *x*-axis, *r*(537) = .28, *p* < .001, BF_10_ = 2.00 × 10^8^, and the *y*-axis, *r*(537) = .31, *p* < .001, BF_10_ = 3.30 × 10^10^ (see Figure 4). Responses were excluded if they were more than 2.5 standard deviations away from the target (3.75 m; 2.9% or 31 observations). Variable error was worse (higher) than the Bayesian optimal variable error along both the E/W axis, *t*(19) = –3.13, *p* = .003, *d* = –.70, BF_10_ = 12.20, and the N/S axis, *t*(19) = –3.87, *p* = .001, *d* = –.87, BF_10_ = 50.47 (see Figure 5). Accuracy was better in NSEW trials versus the NS or EW trials, *t*(19) = –3.30, *p* = .004, *d* = –.74, BF_10_ = 11.84 (see Figure 6). Accuracy was not better in the NSEW trials versus the near trials, *t*(19) = .25, *p* = .803, *d* = .06, BF_01_ = 3.22. By the same logic as that used for the adults, this favors adaptive selection.

### Interim Discussion

The results of Experiment 1 point toward adaptive selection for both adults and children. Adaptive selection is a non-Bayesian process of selecting the best single cue and using it in isolation. For children under 10 years, this is in line with previous research regarding the use of multiple cues (Adams, 2016; Burr & Gori, 2011; Chambers et al., 2018; Dekker et al., 2015; Gori et al., 2012; Jovanovic & Drewing, 2014; Nardini et al., 2008, 2010, 2013; Petrini et al., 2014). Reanalysis of previous data agrees as well. This means that, in regard to the children, we now have a consistent and clear pattern of results. They likely do not use a Bayesian process in the classic geometric reorientation paradigm (see the reanalysis of A vs. A + G conditions). They do not use a Bayesian process in the present paradigm. They do not use a Bayesian process when given landmark and self-motion cues (Nardini et al., 2008). Children under 10 generally do not use multiple cues in a Bayesian manner (Burr & Gori, 2011; though see Negen et al., 2019).

For adults, when considering both the present result and the previous literature, the overall pattern of results is somewhat disjointed and requires further examination. Adults can frequently use a Bayesian process in perception and memory (Pouget et al., 2013). It is not clear why adults would not have used a Bayesian process here. The next experiment is designed to see why this was occurring.

To isolate the variable preventing cue combination, we can closely compare experiment 1 and a previous study that did find cue combination (Jetzschke et al., 2017). Both studies used adults, a virtual reality method, and multiple landmarks as the different cues. However, there are two differences. The previous study did not use an explicit disorientation procedure. Participants were led from a study location to a release location in a circuitous way, but with their eyes open and the landmarks always visible. This makes it difficult to trace the exact route back to the study location but never induces a sense of disorientation. This might be important because disorientation could induce specific neural processes that attend to specific spatial cues more than others (Cheng, 1986; Knierim et al., 1995, 2017). The previous study also used a homing task, asking participants to return to the homing location, rather than a recall task, asking participants to select where a target location was presented. This is potentially important because homing relative to landmarks can be completed in a completely egocentric fashion, just remembering a “snapshot” of what the landmarks looked like from the studied home viewpoint (Stürzl et al., 2008). The task here requires a completely allocentric strategy. Experiment 2 is therefore as similar as possible to Experiment 1, except it also removes the disorientation aspect; it disrupts egocentric vectors to the targets in a way that does not disorient the participant. If cue combination is observed, then the disorientation is likely preventing cue combination. If not, then the difference is likely due to the task itself (homing vs. recall) and its implications in terms of egocentric versus allocentric reasoning.

## Experiment 2

Experiment 2 is an experiment done solely with adults, as similar as possible to Experiment 1 but without disorientation. This was done to test the hypothesis that adults will combine cues in allocentric spatial tasks without disorientation, but not allocentric spatial tasks with disorientation.

### Method

**Figure 7 fig7:**
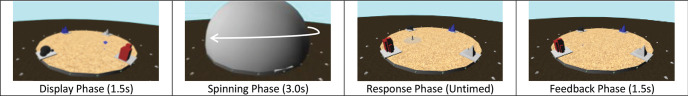
Methods for Experiment 2 *Note*. Participants were shown the target. The target and landmarks were covered and then spun rapidly and erratically. The cover was removed, and the participant would then indicate the target from memory. See the online article for the color version of this figure.

The method was as similar as possible to Experiment 1, except without disorientation (see Figure 7). In short, we spun the target and landmarks instead of the participant. To make this possible, the virtual environment was altered. The target area and the landmarks were raised onto a circular pedestal. The pedestal had identical markers placed around its edge. The ground near the pedestal also had identical markers. There was also a gray half-sphere that could appear over the top of the pedestal, blocking all vision of the target area and the landmarks. The participant’s viewpoint was set back another 2 m so that they could see the spinning platform and the stationary ground around it, making it clear that the platform specifically was spinning (and not the participant moving around it). After being shown the target, the participant was not moved or turned in any way. Instead, the gray half-sphere covered the pedestal. The pedestal spun rapidly and erratically for three seconds, making it impossible to track the target egocentrically. The gray half-sphere was removed. The participant then attempted to point to the target location. This requires the participants to use the landmarks, which is the same as Experiment 1. One might think of this as a local or intrinsic allocentric frame. However, it induces no sense of disorientation. Beyond this, the experiment was the same as Experiment 1.[Fig-anchor fig7]

### Results

**Figure 8 fig8:**
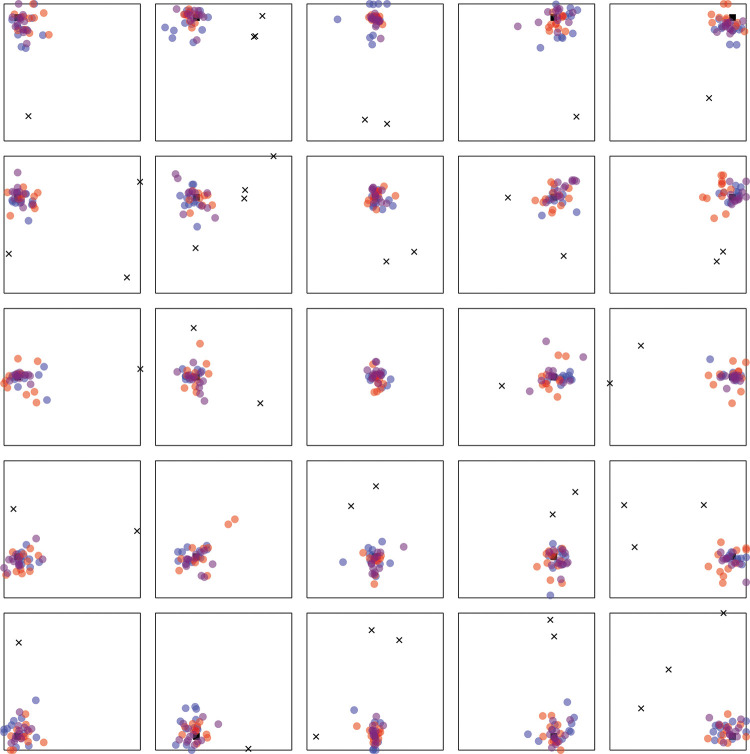
Adult Data From Experiment 2 (Without Disorientation) *Note*. Red dots are responses on NS trials, where the North (N) and South (S) landmark are visible. Blue dots are East West (EW) trials. Purple dots are NSEW trials. The black square is the target. Black crosses are excluded responses. See the online article for the color version of this figure.

**Figure 9 fig9:**
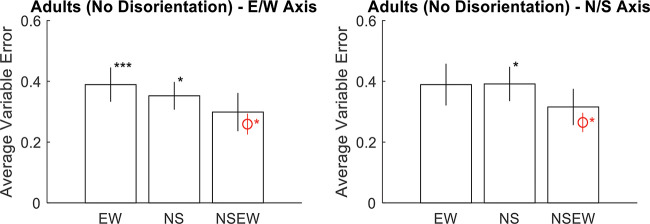
Average Variable Error Broken Down by Trial Type (x-Axis) and Axis of the Space (Left Versus Right Panels) *Note*. Error bars are 95% confidence intervals for the mean. Asterisks mark significant paired *t* tests against NSEW. The red marking is the Bayesian optimal variable error. Both groups, along both axes, had significantly higher (worse) variable error than the Bayesian optimal variable error when shown all landmarks. This speaks against adaptive combination but is consistent with either adaptive selection or random selection. NSEW = North South East West. * *p* < .05. *** *p* < .001. See the online article for the color version of this figure.

**Figure 10 fig10:**
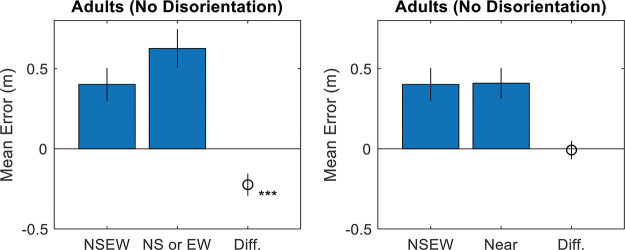
Accuracy Compared With the NSEW Trials, Broken Down by Trial Type (x-Axis) and Comparison Trials (Left Versus Right Panels) *Note*. Results favor adaptive selection, which predicts a difference versus North South (NS) or East West (EW) trials but not versus near trials. *** *p* < .001. See the online article for the color version of this figure.

Results again favor adaptive selection. The responses were correlated with the targets along the *x*-axis, *r*(898) = .91, *p* < .001, BF_10_ = 1.1 × 10^341^, and the *y*-axis, *r*(898) = .90, *p* < .001, BF_10_ = 3.41 × 10^321^ (see Figure 8). Responses were excluded if they were more than 2.5 standard deviations away from the target (1.5 m; 2.6% or 46 observations). Variable error was worse than the Bayesian optimal variable error along both the E/W axis, *t*(11) = –2.15, *p* = .028, *d* = –.62, BF_10_ = 2.18, and the N/S axis, *t*(11) = –1.93, *p* = .040, *d* = –.56, BF_10_ = 1.64 (see Figure 9). Accuracy was better in NSEW trials versus the NS or EW trials, *t*(11) = –7.15, *p* < .001, *d* = –2.06, BF_10_ = 577.52 (see Figure 10). Accuracy was not better in the NSEW trials versus the Near trials, *t*(11) = .30, *p* = .772, *d* = .09, BF_01_ = 2.54. All these patterns are the same as Experiment 1.[Fig-anchor fig8][Fig-anchor fig9][Fig-anchor fig10]

While the results are the same as the adults in Experiment 1 in terms of favoring adaptive selection, the lack of disorientation did lead to better overall performance. In a 2 (disorientation vs. no disorientation) × 2 (NSEW vs NS or EW) ANOVA, using mean error as the dependent variable, there was a significant effect of disorientation, *F*(1, 22) = 4.47, *p* = .046, η^2^ = .124, BF_10_ = 1.83. Similarly, in a 2 (disorientation vs. no disorientation) × 2 (NS axis vs. EW axis) × 3 (NS, EW, or NSEW landmarks visible) ANOVA, using variable error as the dependent variable, there was a main effect of disorientation, *F*(1, 22) = 7.77, *p* = .011, η^2^ = .171, BF_10_ = 2.29, with worse (higher) variable error after disorientation.

The pattern of results above will likely raise post hoc questions about the possibility that participants were using multiple cues in a suboptimal way. In all three samples across both experiments, there were multiple times when the NSEW variable error was significantly lower than the NS or EW variable error. In Experiment 2, the variable error along the E/W axis was significantly lower in NSEW trials than NS trials and also significantly lower than EW trials. In many related studies, this would be taken as evidence for suboptimal cue combination. We examined the data for evidence of suboptimal cue combination and ultimately concluded that there is not sufficient evidence to warrant this interpretation.

To examine this properly, we have to look carefully at the predictions made by adaptive selection. This hypothesis, which does not involve using two cues on the same trial, can still account for a lower variable error in NSEW trials than NS or EW trials. This is because some NS or EW trials have a larger distance from target to landmark than any of the NSEW trials. If these long distances to the landmark increase variable error, then a person who uses the nearest single landmark for encoding would still have a higher variable error in NS or EW trials than NSEW trials. Instead, to show that variable error decreases with additional landmarks in a way that cannot be explained by adaptive selection, we must look at near trials. Adaptive selection predicts that there will not be a difference in variable error between NSEW trials and near trials. Participants in both would just encode against the nearest single landmark. Suboptimal cue combination predicts that variable error will be lower in NSEW trials than Near trials. Participants would integrate the additional information for higher precision.

**Figure 11 fig11:**
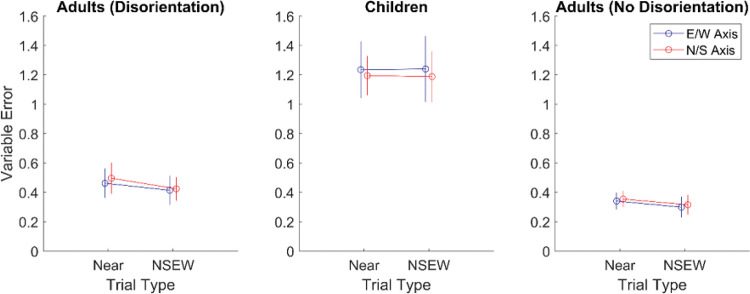
Variable Error as a Function of Trial Type (NSEW or Near), Axis, and Participant Group *Note*. Suboptimal cue combination would predict that variable error in near trials will be higher than variable error in NSEW trials. Adaptive selection predicts that this effect should not appear. This effect was not significant in the present data. Error bars are 95% confidence intervals. NSEW = North South East West. See the online article for the color version of this figure.

To examine this, we calculated the variable error in near trials and NSEW trials separately for each participant. These were entered into a 3 (group: children, adults with disorientation, adults without disorientation) × 2 (trial type: NSEW or near) × 2 (axis: N/S or E/W) mixed ANOVA (see Figure 11). The main effect of trial type was not significant, *F*(1, 41) = .69, *p* = .41, η^2^ = .001, BF_01_ = 13.64, meaning that variable error was not significantly higher in near trials than NSEW trials. No other within-subjects effects or interactions were significant. As expected, there was a main effect of group, with children having the highest variable error and the adults without disorientation having the lowest, *F*(2, 41) = 80.8, *p* < .001, η^2^ = .69, BF_10_ = 6 × 10^11^. A Friedman test was also conducted due to potential issues with unequal variance, entering NSEW N/S, NSEW E/W, near E/W, and near N/S variable errors. This did not find any effect, χ^2^(3) = 4.34, *p* = .23. All of this fails to support suboptimal cue combination over Adaptive Selection; if anything, the BF result (BF_01_ = 13.64) points toward the lack of suboptimal cue combination.[Fig-anchor fig11]

This might also bring up some questions about our optimality predictions, so please allow us to present some short theoretical results to clarify that the optimal predictions are not biased toward Adaptive Selection. The typical formulation requires variable error to be constant for all targets (Rohde et al., 2016), which seems to be violated in the present study. This makes it possible to achieve a dual-cue variable error that is below (better than) the optimal prediction calculated here. This is because the optimal prediction uses a particular kind of average over targets (root mean squared), but some targets will have variable error below the average variable error, which creates a kind of lever for deeper noise reductions. Specific numbers will help as an example. Suppose Cue 1 has a variance of 1 at Location A and 2 at Location B. Suppose Cue 2 is the opposite, having a variance of 2 at Location A and 1 at Location B. The overall variance of each cue will be measured on average at 1.5 (i.e., [1 + 2]/2 = 1.5). The optimal prediction will be three fourths (i.e., [1.5^−1^ + 1.5^−1^]^−1^ = 3/4). The true optimal at each location will be two thirds (i.e., [1^−1^+2^−1^]^−1^ = 2/3). As two thirds is less than three fourths, a truly optimal process could do better than the estimated optimal and a suboptimal process could meet the estimated optimal. This would bias the results toward optimal cue combination. Although differences in variable error due to distance to the nearest target could result in some small issues with the accuracy of our optimality predictions, these inaccuracies are expected only to go against our theoretical conclusion here.

### Interim Discussion

Experiment 2 was done to see if the difference in results between adults in Experiment 1 and a previous study (Jetzschke et al., 2017) was due to the use of disorientation in Experiment 1. Because results were like Experiment 1 (i.e., not showing cue combination), but Experiment 2 did not involve disorientation, this hypothesis seems unlikely. Instead, this isolates a more fundamental aspect of the tasks: Here, participants had to use landmarks in a local allocentric frame to recall locations, whereas the previous study asked participants to return “home” in a way that allows egocentric snapshots to be useful. Other than this, Experiment 2 and the previous study both tested adults, used virtual methods, did not disorient participants, and used multiple landmarks as the cues.

It may also be helpful to contrast the difference in Experiments 1 and 2 versus other studies that use movement of observer versus scene. Moving a participant around a scene often results in better performance than moving a scene in front of a participant (e.g., Mou et al., 2009). Here, changing the participant’s viewpoint within a stable scene led to worse performance than moving the scene in front of the participant. There could be several reasons for the contrast. The most obvious is that vestibular information could be used to update egocentric relations to the scene in other studies; moving the participant might allow for a more accurate egocentric strategy that was not available in Experiment 1 here. This also fits with a series of additional findings where the advantage is eliminated or reversed by giving participants additional information about the magnitude of the displacement in lieu of vestibular information (Mou et al., 2009).

## General Discussion

Both experiments point strongly toward adaptive selection, a non-Bayesian process of selecting the most useful landmark and using it isolation. They point away from adaptive combination, a Bayesian process. Specifically, the Bayesian predictions about precision were consistently violated. They also point away from random selection, a non-Bayesian process of selecting a landmark to use at random. Specifically, accuracy was better than we would expect from using a random landmark. In contrast, results are consistent with all three predictions if participants are just encoding the target location against the nearest single landmark. We interpret this to mean that landmarks are not used together in a Bayesian fashion to recall locations, at least in a situation where egocentric relations have been disrupted; instead, people use the nearest available landmark to code locations. This provides an immediate theoretical point: that the adaptive combination model, taken as a general theory of how multiple cues are used to reorient, is not as broadly applicable as one might have hoped. We propose considering the older adaptive selection model, which still allows young children to use superior cues in place of inferior cues when both are available, but not to use superior cues in Bayesian combination with inferior cues.

To aid in interpretation, we need to point out a few things about the current study. Our focus was not particularly on the way that boundaries, including rectangular boundaries, are used to reorient. Instead, the goal of the design here was to find a situation where the predictions of a Bayesian cue combination model for reorientation could be clearly confirmed or discredited. Under our reading, adaptive combination is intended to be a flexible framework for the way that any set of valid cues are used to reorient—not just rectangular enclosures. To make the predictions of this framework as clear as possible in the present study, we used pairs of landmarks as cues. The results here speak against the general form of the adaptive combination model (especially Equation 1) as a way for any reorientation cues to combine for allocentric recall. For a researcher who is specifically interested in the use of rectangular enclosures, rather than a general theory of how reorientation happens, the new data presented here have a more modest interpretation. It could still be the case that other cues are used in a Bayesian fashion to reorient, perhaps even at young ages. We suggest holding off on that conclusion unless and until more evidence for it is found.

We should also point out that a variation without disorientation did not appear to alter results. In other words, these results do not appear strictly limited to reorientation. Instead, they appear to apply to situations where egocentric relations are broken. It does appear that using landmarks to reorient is a non-Bayesian process, but it may make more sense to describe this in terms that are more general: Landmarks are not used in a Bayesian process to recall locations when the use of the allocentric frame is forced.

Our reanalysis of previous data also suggests that geometric and associative cues are not combined in a Bayesian fashion by young children, but here we must be more tentative. In the introduction, we reanalyzed previous data to compare performance in an associative-only reorientation task versus an A + G (one uniquely colored wall in a rectangle) reorientation task. No difference was found. This does not fit well with the idea that the associative cue’s information is being combined in the optimal Bayesian manner with the geometric cue’s information. Instead, it suggests that the associative cue’s information is used in isolation. However, this analysis is far from ideal. For example, it uses between-subjects data. In our view, this specific question remains open.

It should also be pointed out that adaptive combination and adaptive selection can make nearly identical predictions in the right circumstances. For example, pose a child is given a very strong associative cue (e.g., a very salient and nonsymmetric picture on one wall) and a very weak geometric cue (e.g., a rectangular boundary with a length of 2 m and a width of 2.05 m). Adaptive selection would select the associative cue and the child would perform as if they only had the associative cue. Adaptive combination would weight the two cues together according to Equation 1, but because the geometric cue is much weaker, it would receive negligible weight, and the results would not be measurably different to those based on using the associative cue alone. In general, the two theories make very similar predictions in any situation with one dominant reorientation cue. Differences can only become clear when there are multiple reorientation cues with comparable reliability.

As far as we are aware, the present interpretation of an A + G condition is novel. In the developmental literature, it is well established that young children can use purely geometric cues to reorient (Lee, 2017). In interpreting the results of an A + G condition, the usual question has been whether the associative cue is used in concert with the geometric cue (Cheng et al., 2013; Hermer & Spelke, 1994). It could be the case that the associative cue is used in isolation while ignoring the geometric cue—at least in situations with a relatively large room. (In a small room, in contrast, it is well established that performance is similar to only having the geometric cue.) It may be possible to test an exclusive reliance on the associative cue more directly in the future, but it would require some significant methodological innovations. Ideally, the same participants would complete a large number of A, G, and A + G condition trials. It is not obvious how to make the standard paradigm into something that will be tolerated by young children for significantly longer. Further, details of the method would need to be adjusted somehow to make A performance better-matched to G performance—perhaps by reducing the contrast of the associative cue and exaggerating the ratio of the rectangle’s lengths. Further, and perhaps most difficult, it is not clear how this kind of paradigm would differentiate Bayesian reasoning from other simpler models. For example, the information from the geometric and associative cue could be combined through conjunctive logic (e.g., search until finding a target that agrees with both remembered cues) rather than probabilistic Bayesian reasoning. This would also predict that A + G performance would be better than A performance. It may ultimately be more fruitful to move to new paradigms.

To be as fair as possible to our colleagues (Xu et al., 2017), we should also note that the adaptive combination article did not explicitly state its intention to apply to tasks with multiple landmarks (i.e., associative cues). Under our reading, they put forward a theory that tries to unify reorientation behavior under single compact principle. Because this was a Bayesian model, we would ordinarily expect it to efficiently integrate all available cues—that is such a core feature of such models that it practically serves as a definition—and that this would include multiple associative cues. (They also did not say that it does not apply to multiple associative cues.) If the reader here disagrees with our reading of the adaptive combination model, then the present study should be taken as an examination of general Bayesian principles in reorientation rather than a specific reexamination of the adaptive combination model.

We should emphasize that adaptive combination is a recent extension of the adaptive behavior position; rejecting adaptive combination does entail rejecting all adaptive explanations of how young children reorient. It should not be interpreted to mean that a modular theory (Hermer & Spelke, 1994), the usual contrast to an adaptive theory, should be preferred. It would require a very different kind of experiment to potentially show evidence for nonadaptive and modular cognition (Lee, 2017; Lee & Spelke, 2008, 2010). Instead, adaptive selection is more in line with the versions of adaptive theories proposed before adaptive combination (Cheng et al., 2013). In addition, while our results speak against adaptive combination as a general theory of spatial reorientation, it remains possible that it does apply to some situations—for example, recall that can include an egocentric process of homing (Jetzschke et al., 2017; Stürzl et al., 2008), or recall using other kinds of spatial cues (although see above on difficulties of testing these in a Bayesian framework).

We should also note, in case there is any doubt, that adaptive selection can also explain all of the data cited by the adaptive combination article as well (Hermer & Spelke, 1996; Learmonth et al., 2002; Newcombe et al., 2010; Ratliff & Newcombe, 2008a,’ 2008b). This is simply because none of them test a condition with one cue alone, with another cue alone, and with both together. Most specifically test A + G conditions against G conditions. This always allows for performance in the A + G condition to be explained by use of the A cue alone. The others vary but can be explained with a similar argument, such as comparing an A + G condition to a condition with a language cue added (Ratliff & Newcombe, 2008a). Without individually testing the A cue, the G cue, and the language cue, it is impossible to rule out the hypothesis that performance in the combined condition is reliant on just one of the cues.

One interesting future direction would be to test the efficiency of cue selection in a setting where there are not just landmarks. If one landmark is further than another is, it is fairly clear that it will be less useful for encoding. If a young participant is asked to choose among a more diverse set of cues (e.g., including a linguistic cue), it is not yet known if they will consistently select the most useful cue.

Another interesting future direction is to look more at the potential role of cue salience. The present study and the standard reorientation paradigm both use environments that are (much) less rich than many environments that exist outside the laboratory. It is possible that these processes would be meaningfully different in an environment with a great many visual cues, additional strong sensations, and a complex geometry. It is possible that participants might integrate multiple cues in a very rich and naturalistic environment as a way of compensating for the increased memory noise that such complexity would induce. It is also possible that participants might integrate multiple cues if their salience is greatly increased in comparison to the rest of the environment. In a selection model, things like visual salience (rather than distance to target) might be important for understanding cue selection.

## Toward a More General Theory

Here we outline how these results may drive us toward a more general theory of how reorientation happens with multiple cues. The present study provides an immediate empirical conclusion: children and adults select the nearest landmark to use in isolation for encoding targets during an allocentric reorientation task. A larger interpretive framework will require more research. To move this forward, we will sketch one plausible model (of many) to pursue and test further. In short, we consider that egocentric spatial information may typically be treated in a Bayesian manner after a certain point in development, sometime in middle childhood; allocentric information, across the life span, may instead be processed with more idiosyncratic non-Bayesian heuristics.

That idea has three parts. First, egocentric spatial information is typically used in a Bayesian manner by adults. This fits in a variety of very simple perceptual tasks where participants are asked to make judgements about locations. For example, adults can combine a spatialized sound and a noisy visual cue to judge horizontal location in an egocentric frame (Battaglia et al., 2003; Gori et al., 2012). This also applies to newly-learned skills that signal egocentric distance, such as an echolocation-like skill taught over the course of a few hours (Negen, Wen, et al., 2018). This further applies in a navigation task where the two cues are vestibular and proprioceptive (Frissen et al., 2011). Adults also rapidly learn egocentric (sensorimotor) prior distributions and use them in a Bayesian fashion as well (Bejjanki et al., 2016; Berniker et al., 2010; Chambers et al., 2018; Körding & Wolpert, 2004; Kwon & Knill, 2013; Narain et al., 2013; Sato & Kording, 2014; Tassinari et al., 2006). In practice, this means that they learn where targets tend to be and bias their responses toward the places they tend to be most often. Finally, adults tend to adjust their search strategy in a visual search task when there is an uneven distribution of targets in an egocentric sense (Jiang & Swallow, 2013, 2014; Smith et al., 2010). This can be viewed as using egocentric prior distributions to affect decision-making.

Importantly, one could view self-motion and landmark information in a homing task as egocentric information. These are the cues and the task used in a series of studies where adults were fit well by a Bayesian model (Bates & Wolbers, 2014; Chen et al., 2017; Nardini et al., 2008; Sjolund et al., 2018; Zhao & Warren, 2015). The self-motion information could be viewed as an egocentric vector to the goal that is updated by perception of own movement. The landmark information, in this case, could be like an egocentric “snapshot” of how the landmarks looked at the target (home) location (Cheung et al., 2008; Stürzl et al., 2008). In other words, while landmarks are usually thought of as allocentric information, the specific way that landmarks looked from a previous home location could be stored in an egocentric format. This makes this finding fit with the idea that adults use egocentric information in a Bayesian fashion.

Of course, recent research has shown that this also faces some limits and suboptimalities (Rahnev & Denison, 2018)—many Bayesian processes are distorted under certain circumstances. For example, in one study, adults integrated multiple repeats of the same audio localization signal with lower than Bayesian efficiency (Jones, 2018). It is not clear exactly why this occurred, but adults’ performance was much nearer to optimal Bayesian integration when the signals were not exact repeats of each other. We should emphasize that it may be typical for egocentric information to be processed in a Bayesian way, but it will not be universal.

Second, egocentric spatial information is not used in a Bayesian manner by young children. This would explain their difficulty in making egocentric spatial judgements with an audio and a visual cue (Gori et al., 2012), difficulty combining self-motion and landmark information during a homing task (Nardini et al., 2008), and their difficulty in learning prior spatial distributions (Chambers et al., 2018). This fits more generally with the pattern of difficulty with Bayesian reasoning in a wide variety of settings (Burr & Gori, 2011).

Third, allocentric spatial information is not used in a Bayesian manner. This fits with all the findings here. Instead, what the participants did here appears to involve focusing on “just enough” of the allocentric spatial relations to uniquely encode the target location in principle. It could be that once a task involves allocentric computations, capacity for the number of cues that can be attended to becomes a major bottleneck. At that point, it may be more advantageous to focus attention on the way that a target relates to a single nearby landmark than to spread attention across an entire scene. This also fits with visual search patterns in an allocentric frame. Participants do not tend to use the prior distribution to adjust their search strategies (Jiang & Swallow, 2013, 2014; Smith et al., 2010). This is, again, “just enough”—learning the prior distribution does not affect the participant’s ability to complete the task; it only makes it faster to do so. It may be that allocentric reasoning is too slow for it to affect the way the visual search task is completed. In general, the complexities of the representations required for allocentric reasoning may be too slow and too costly to be a good application of Bayesian reasoning. That may be reserved instead for mature egocentric reasoning.

## Conclusion

The present study was designed further test predictions from the adaptive cue combination model of human spatial reorientation, understood here as a general model of how multiple cues are used to retrieve vectors to goal locations after losing one’s sense of heading and placement in a space. The results suggest that this theory needs modification since the optimal Bayesian predictions were consistently violated. Instead, response patterns were more consistent with a heuristic of only using the nearest single landmark (ignoring other landmarks rather than combining their information in a Bayesian fashion). Further, results are similar if egocentric relations are disrupted through a method without disorientation. As a sketch of a broader theory for further testing, we suggest that egocentric information may typically be used with Bayesian efficiency after middle childhood (emerging from roughly 7 to 12 years depending on task details), but that allocentric information is processed using non-Bayesian heuristics even into adulthood.

## Supplementary Material

10.1037/xhp0000950.supp

## Figures and Tables

**Figure A1 fig12:**
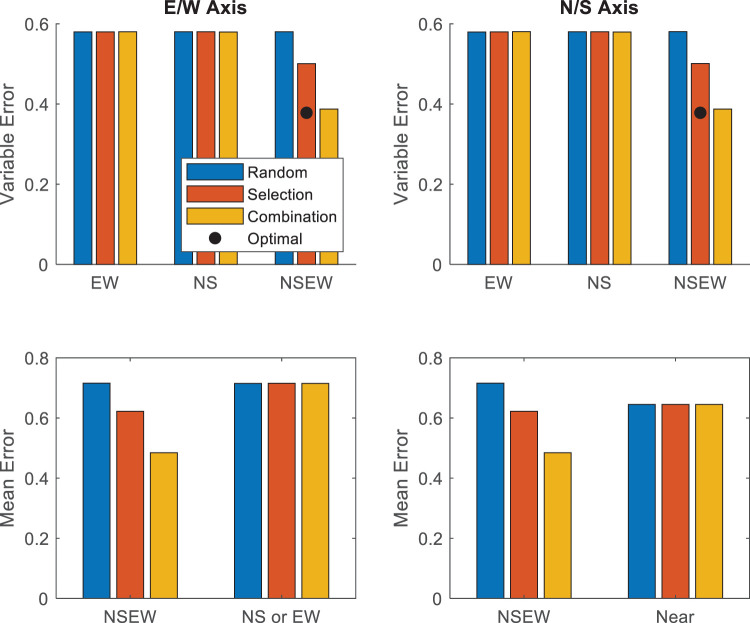
Simulation Results *Note*. See the online article for the color version of this figure.

## Footnotes

1 In the original studies (Cheng, 1986), the method was to gently remove the animal, move the experimental apparatus to another part of the room, then gently replace the animal with a new viewpoint and direction, all in the dark. There was no spinning or attempt to induce a vestibular/proprioceptive signal related to the disorientation. In subsequent studies with humans (particularly, children), practicalities including time limits made it necessary to induce viewpoint change via blindfolding/spinning. This has some unfortunate side effects, including the possibility of disturbing the young participants and making them feel dizzy. We consider virtual viewpoint changes to be a welcome return to avoiding these unwanted issues, if participants are clearly made aware in other ways (as here) that a viewpoint change has occurred.

## Model Simulations

To be as sure as possible about the predictions, we also simulated each model. The MATLAB (MathWorks, 2019) code is shown in following figure. We assume that the precision of a single-cue response decays exponentially with the distance to the nearest target. Results are shown in Figure A1, depicting the random model [blue/left bars]) chooses which cue to use as at random for dual-cue trials. The selection model (see Figure A1, orange/middle bars) chooses the single cue with the highest precision for dual-cue trials. The combination model (yellow/right bars) creates a precision-weighted average on dual-cue trials. The top two rows of Figure A1 show that only the Combination model achieves the optimal variable error (black dot). The bottom left shows that NSEW accuracy equals NS or EW accuracy for the random model, but NSEW accuracy is better than NS or EW accuracy for the other two models. The bottom right shows that NSEW accuracy is worse than near accuracy for random, equal for selection, and better for combination. Note how the patterns in the orange/middle bars are the same as the actual data.[Fig-anchor fig12]

Here is the simulation code: Matlab (MathWorks, 2019):
%Size of simulation and parameters
nDataPerTarget = 100000; 
EWLandmarksX = [3, −3]; 
EWLandmarksY = [0, 0]; 
NSLandmarksX = [0, 0]; 
NSLandmarksY = [3, −3]; 
[X,Y] = meshgrid(−2:2, −2:2); 
Targets = [X(:),Y(:)]; 
%Mechanism for decay of precision by distance
BasePrecision = 10; 
PrecisionDecayBeta = .5;
DistanceToPrecision = @(distance) BasePrecision .* exp(-PrecisionDecayBeta.*distance);

%Pre-Calculate precisions or each target
PrecisionEW = NaN(size(Targets,1),1); 
PrecisionNS = NaN(size(Targets,1),1); 
for i = 1:length(PrecisionEW)
    DistanceE = sqrt((Targets(i,1)-EWLandmarksX(1)).^2 + (Targets(i,2)-EWLandmarksY(1)).^2);
    DistanceW = sqrt((Targets(i,1)-EWLandmarksX(2)).^2 + (Targets(i,2)-EWLandmarksY(2)).^2);
    PrecisionEW(i) = DistanceToPrecision(min(DistanceE,DistanceW));

    DistanceN = sqrt((Targets(i,1)-NSLandmarksX(1)).^2 + (Targets(i,2)-NSLandmarksY(1)).^2);
    DistanceS = sqrt((Targets(i,1)-NSLandmarksX(2)).^2 + (Targets(i,2)-NSLandmarksY(2)).^2);
    PrecisionNS(i) = DistanceToPrecision(min(DistanceN,DistanceS)); 
end
StandardDevEW = PrecisionEW .^ (−1/2); 
StandardDevNS = PrecisionNS .^ (−1/2);

%Simulate trials
%Pre-allocate
target = NaN(nDataPerTarget*size(Targets,1)*3,2);
response = target; 
isNear = false(nDataPerTarget*size(Targets,1)*3,1);
isNSEW = isNear; isNSorEW = isNear; isEW = isNear; isNS = isNear;  
%Simulate
for modelNumber = 1:3
  IND = 1;
  for repeat = 1:nDataPerTarget
    for targetNumber = 1:size(Targets,1)
      for trialType = 1:3 %Trial types: EW, NS, NSEW

        target(IND,:) = Targets(targetNumber,:);
               isNear(IND,1) = (trialType==1 && abs(target(IND,1))>=abs(target(IND,2))) ‖ …
          (trialType==2 && abs(target(IND,2))>=abs(target(IND,1)));
      isNSEW(IND,1) = trialType == 3;
      isNSorEW(IND,1) = trialType ∼= 3;
      isEW(IND,1) = trialType == 1;
      isNS(IND,1) = trialType == 2;
      if trialType == 1 %EW Trial
              response(IND,:) = target(IND,:) + randn(1,2) .*StandardDevEW(targetNumber);
      elseif trialType == 2 %NS Trial
              response(IND,:) = target(IND,:) + randn(1,2) .*StandardDevNS(targetNumber);
      else %NSEW Trial
        if modelNumber == 1 %Random
          if rand(1) < .5
              response(IND,:) = target(IND,:) + randn(1,2) .*StandardDevEW(targetNumber);
          else
              response(IND,:) = target(IND,:) + randn(1,2) .*StandardDevNS(targetNumber);
          end
        elseif modelNumber == 2 %Selection
              response(IND,:) = target(IND,:) + randn(1,2) .*min(StandardDevEW(targetNumber),StandardDevNS(targetNumber));
        else %Combination
                 tmpEW = target(IND,:) + randn(1,2) .* StandardDevEW(targetNumber);
                 tmpNS = target(IND,:) + randn(1,2) .* StandardDevNS(targetNumber);
                 wEW = PrecisionEW(targetNumber) / (PrecisionEW(targetNumber)+PrecisionNS(targetNumber));
          wNS = 1 - wEW;
              response(IND,:) = tmpEW.*wEW + tmpNS.*wNS;
        end
      end

      IND = IND + 1;

     end
   end
 end    
 %Produce summaries

 VariableErrorEW(modelNumber,1) = std(response(isEW,1)  -target(isEW,1));
  VariableErrorEW(modelNumber,2) = std(response(isNS,1)  -target(isNS,1));
       VariableErrorEW(modelNumber,3) = std(response(isNSEW,1)-target(isNSEW,1));

  VariableErrorNS(modelNumber,1) = std(response(isEW,2)  -target(isEW,2));
  VariableErrorNS(modelNumber,2) = std(response(isNS,2)  -target(isNS,2));
       VariableErrorNS(modelNumber,3) = std(response(isNSEW,2)-target(isNSEW,2));

     NSEWAccuracy(modelNumber,1) = mean(sqrt((target(isNSEW,1)-response(isNSEW,1)).^2 + … (target(isNSEW,2)-response(isNSEW,2)).^2));
     NSorEWAccuracy(modelNumber,1) = mean(sqrt((target(isNSorEW,1)-response(isNSorEW,1)).^2 + … (target(isNSorEW,2)-response(isNSorEW,2)).^2));
       NearAccuracy(modelNumber,1) = mean(sqrt((target(isNear,1)-response(isNear,1)).^2 + … (target(isNear,2)-response(isNear,2)).^2));

 end

 %Display results
 %EW VE
 subplot(2,2,1); hold on
 h = bar(VariableErrorEW');
 ylabel('Variable Error')
 set(gca,'XTick',1:3)
 set(gca,'XTickLabel',{'EW','NS','NSEW'})
 plot(3, (mean(mean(VariableErrorEW(:,2:3))).^-2.*2).^(-1/2),
 'ko', 'MarkerFaceColor','k')
 title('E/W Axis')
 legend
 ('Random','Selection','Combination','Optimal','location','SouthEast')

 %NS VE
 subplot(2,2,2); hold on
 bar(VariableErrorNS')
 ylabel('Variable Error')
 set(gca,'XTick',1:3)
 set(gca,'XTickLabel',{'EW','NS','NSEW'})
 plot(3, (mean(mean(VariableErrorNS(:,2:3))).^-2.*2).^(-1/2), 'ko',
 'MarkerFaceColor','k')
 title('N/S Axis')

 %NSEW vs NSorEW Accuracy
 subplot(2,2,3)
 bar([NSEWAccuracy, NSorEWAccuracy]')
 set(gca,'XTick',1:3)
 set(gca,'XTickLabel',{'NSEW','NS or EW'})
 ylabel('Mean Error')

 %NSEW vs Near Accuracy
 subplot(2,2,4)
 bar([NSEWAccuracy, NearAccuracy]')
 set(gca,'XTick',1:3)
 set(gca,'XTickLabel',{'NSEW','Near'})
 ylabel('Mean Error')

## References

[c1] Adams W. J. (2016). The development of audio–visual integration for temporal judgments. PLoS Computational Biology, 12(4), e1004865. 10.1371/journal.pcbi.1004865 27078834 PMC4831745

[c2] Bates S. L., & Wolbers T. (2014). How cognitive aging affects multisensory integration of navigational cues. Neurobiology of Aging, 35(12), 2761–2769. 10.1016/j.neurobiolaging.2014.04.003 24952995

[c3] Battaglia P. W., Jacobs R. A., & Aslin R. N. (2003). Bayesian integration of visual and auditory signals for spatial localization. Journal of the Optical Society of America. A, Optics, Image Science, and Vision, 20(7), 1391–1397. 10.1364/JOSAA.20.001391 12868643

[c4] Bejjanki V. R., Knill D. C., & Aslin R. N. (2016). Learning and inference using complex generative models in a spatial localization task. Journal of Vision, 16(5), 9. 10.1167/16.5.9 PMC4790422 26967015

[c5] Berniker M., Voss M., & Kording K. (2010). Learning priors for Bayesian computations in the nervous system. PLoS ONE, 5(9), e12686. 10.1371/journal.pone.0012686 20844766 PMC2937037

[c6] Burr D., & Gori M. (2011). Multisensory integration develops late in humans. In Murray M. M. & Wallace M. T. (Eds.), The neural bases of multisensory processes (pp. 345–362). CRC Press/Taylor & Francis. 10.1201/9781439812174-23 22593886

[c7] Chambers C., Sokhey T., Gaebler-Spira D., & Kording K. P. (2018). The development of Bayesian integration in sensorimotor estimation. Journal of Vision, 18(12), 8. 10.1167/18.12.8 PMC6241171 30452586

[c8] Chen X., McNamara T. P., Kelly J. W., & Wolbers T. (2017). Cue combination in human spatial navigation. Cognitive Psychology, 95, 105–144. 10.1016/j.cogpsych.2017.04.003 28478330

[c9] Cheng K. (1986). A purely geometric module in the rat’s spatial representation. Cognition, 23(2), 149–178. 10.1016/0010-0277(86)90041-7 3742991

[c10] Cheng K., Huttenlocher J., & Newcombe N. S. (2013). 25 years of research on the use of geometry in spatial reorientation: A current theoretical perspective. Psychonomic Bulletin & Review, 20(6), 1033–1054. 10.3758/s13423-013-0416-1 23456412

[c11] Cheung A., Stürzl W., Zeil J., & Cheng K. (2008). The information content of panoramic images II: View-based navigation in nonrectangular experimental arenas. Journal of Experimental Psychology: Animal Behavior Processes, 34(1), 15–30. 10.1037/0097-7403.34.1.15 18248112

[c12] Cressant A., Muller R. U., & Poucet B. (1997). Failure of centrally placed objects to control the firing fields of hippocampal place cells. The Journal of Neuroscience: The Official Journal of the Society for Neuroscience, 17(7), 2531–2542. 10.1523/JNEUROSCI.17-07-02531.1997 9065513 PMC6573492

[c13] Dekker T. M., Ban H., van der Velde B., Sereno M. I., Welchman A. E., & Nardini M. (2015). Late development of cue integration is linked to sensory fusion in cortex. Current Biology, 25(21), 2856–2861. 10.1016/j.cub.2015.09.043 26480841 PMC4635311

[c14] Doeller C. F., & Burgess N. (2008). Distinct error-correcting and incidental learning of location relative to landmarks and boundaries. Proceedings of the National Academy of Sciences of the United States of America, 105(15), 5909–5914. 10.1073/pnas.0711433105 18413609 PMC2311326

[c15] Ernst M. O., & Banks M. S. (2002). Humans integrate visual and haptic information in a statistically optimal fashion. Nature, 415(6870), 429–433. 10.1038/415429a 11807554

[c16] Frissen I., Campos J. L., Souman J. L., & Ernst M. O. (2011). Integration of vestibular and proprioceptive signals for spatial updating. Experimental Brain Research, 212(2), 163–176. 10.1007/s00221-011-2717-9 21590262

[c17] Gori M., Sandini G., & Burr D. (2012). Development of visuo-auditory integration in space and time. Frontiers in Integrative Neuroscience, 6, 77. 10.3389/fnint.2012.00077 23060759 PMC3443931

[c19] Hermer L., & Spelke E. S. (1994). A geometric process for spatial reorientation in young children. Nature, 370(6484), 57–59. 10.1038/370057a0 8015605

[c300] Hermer L., & Spelke E. (1996). Modularity and development: The case of spatial reorientation. Cognition, 61(3), 195–232. 10.1016/S0010-0277(96)00714-7 8990972

[c20] Hermer-Vazquez L., Moffet A., & Munkholm P. (2001). Language, space, and the development of cognitive flexibility in humans: The case of two spatial memory tasks. Cognition, 79(3), 263–299. 10.1016/S0010-0277(00)00120-7 11165214

[c21] Ito H. T., Zhang S.-J., Witter M. P., Moser E. I., & Moser M.-B. (2015). A prefrontal-thalamo-hippocampal circuit for goal-directed spatial navigation. Nature, 522(7554), 50–55. 10.1038/nature14396 26017312

[c200] Jamovi. (2021). Jamovi (Version 1.8.1) [Computer software]. https://jamovi.org

[c22] Jetzschke S., Ernst M. O., Froehlich J., & Boeddeker N. (2017). Finding home: Landmark ambiguity in human navigation. Frontiers in Behavioral Neuroscience, 11, 132. 10.3389/fnbeh.2017.00132 28769773 PMC5513971

[c23] Jiang Y. V., & Swallow K. M. (2013). Spatial reference frame of incidentally learned attention. Cognition, 126(3), 378–390. 10.1016/j.cognition.2012.10.011 23287419

[c24] Jiang Y. V., & Swallow K. M. (2014). Changing viewer perspectives reveals constraints to implicit visual statistical learning. Journal of Vision, 14(12), 3. 10.1167/14.12.3 PMC4189525 25294640

[c25] Jones P. R. (2018). The development of perceptual averaging: Efficiency metrics in children and adults using a multiple-observation sound-localization task. The Journal of the Acoustical Society of America, 144(1), 228–241. 10.1121/1.5043394 30075655

[c26] Jovanovic B., & Drewing K. (2014). The influence of intersensory discrepancy on visuo-haptic integration is similar in 6-year-old children and adults. Frontiers in Psychology, 5, 57. 10.3389/fpsyg.2014.00057 24523712 PMC3906500

[c201] Kass R. E., & Raftery A. E. (1995). Bayes factors. Journal of the American Statistical Association, 90(430), 773. 10.2307/2291091

[c28] Keinath A. T., Julian J. B., Epstein R. A., & Muzzio I. A. (2017). Environmental geometry aligns the hippocampal map during spatial reorientation. Current Biology, 27(3), 309–317. 10.1016/j.cub.2016.11.046 28089516 PMC5296211

[c29] Knierim J. J., Kudrimoti H. S., & McNaughton B. L. (1995). Place cells, head direction cells, and the learning of landmark stability. The Journal of Neuroscience: The Official Journal of the Society for Neuroscience, 15(3, Pt. 1), 1648–1659. 10.1523/JNEUROSCI.15-03-01648.1995 7891125 PMC6578145

[c30] Körding K. P., & Wolpert D. M. (2004). Bayesian integration in sensorimotor learning. Nature, 427(6971), 244–247. 10.1038/nature02169 14724638

[c31] Kwon O.-S., & Knill D. C. (2013). The brain uses adaptive internal models of scene statistics for sensorimotor estimation and planning. Proceedings of the National Academy of Sciences of the United States of America, 110(11), e1064–e1073. 10.1073/pnas.1214869110 23440185 PMC3600457

[c32] Learmonth A. E., Nadel L., & Newcombe N. S. (2002). Children’s use of landmarks: Implications for modularity theory. Psychological Science, 13(4), 337–341. 10.1111/j.0956-7976.2002.00461.x 12137136

[c33] Lee S. A. (2017). The boundary-based view of spatial cognition: A synthesis. Current Opinion in Behavioral Sciences, 16, 58–65. 10.1016/j.cobeha.2017.03.006

[c34] Lee S. A., Winkler-Rhoades N., & Spelke E. S. (2012). Spontaneous reorientation is guided by perceived surface distance, not by image matching or comparison. PLoS ONE, 7(12), e51373. 10.1371/journal.pone.0051373 23251511 PMC3520913

[c35] Lee S. A., & Spelke E. S. (2008). Children’s use of geometry for reorientation. Developmental Science, 11(5), 743–749. 10.1111/j.1467-7687.2008.00724.x 18801130

[c36] Lee S. A., & Spelke E. S. (2010). A modular geometric mechanism for reorientation in children. Cognitive Psychology, 61(2), 152–176. 10.1016/j.cogpsych.2010.04.002 20570252 PMC2930047

[c202] MathWorks. (2019). Matlab [Computer software]. https://mathworks.com

[c37] Miller N. (2009). Modeling the effects of enclosure size on geometry learning. Behavioural Processes, 80(3), 306–313. 10.1016/j.beproc.2008.12.011 20522319

[c38] Mou W., Zhang H., & McNamara T. P. (2009). Novel-view scene recognition relies on identifying spatial reference directions. Cognition, 111(2), 175–186. 10.1016/j.cognition.2009.01.007 19281971 PMC2703187

[c39] Mou W., & Zhou R. (2013). Defining a boundary in goal localization: Infinite number of points or extended surfaces. Journal of Experimental Psychology: Learning, Memory, and Cognition, 39(4), 1115–1127. 10.1037/a0030535 23088544

[c40] Narain D., van Beers R. J., Smeets J. B. J., & Brenner E. (2013). Sensorimotor priors in nonstationary environments. Journal of Neurophysiology, 109(5), 1259–1267. 10.1152/jn.00605.2012 23235999

[c41] Nardini M., Bedford R., & Mareschal D. (2010). Fusion of visual cues is not mandatory in children. Proceedings of the National Academy of Sciences of the United States of America, 107(39), 17041–17046. 10.1073/pnas.1001699107 20837526 PMC2947870

[c42] Nardini M., Begus K., & Mareschal D. (2013). Multisensory uncertainty reduction for hand localization in children and adults. Journal of Experimental Psychology: Human Perception and Performance, 39(3), 773–787. 10.1037/a0030719 23163790

[c43] Nardini M., Jones P., Bedford R., & Braddick O. (2008). Development of cue integration in human navigation. Current Biology, 18(9), 689–693. 10.1016/j.cub.2008.04.021 18450447

[c44] Nardini M., Thomas R. L., Knowland V. C. P., Braddick O. J., & Atkinson J. (2009). A viewpoint-independent process for spatial reorientation. Cognition, 112(2), 241–248. 10.1016/j.cognition.2009.05.003 19501349

[c45] Negen J., Chere B., Bird L. A., Taylor E., Roome H. E., Keenaghan S., Thaler L., & Nardini M. (2019). Sensory cue combination in children under 10 years of age. Cognition, 193, 104014. 10.1016/j.cognition.2019.104014 31302529

[c46] Negen J., Heywood-Everett E., Roome H. E., & Nardini M. (2018). Development of allocentric spatial recall from new viewpoints in virtual reality. Developmental Science, 21(1), e12496. 10.1111/desc.12496 28256025

[c47] Negen J., Roome H. E., Keenaghan S., & Nardini M. (2018). Effects of two-dimensional versus three-dimensional landmark geometry and layout on young children’s recall of locations from new viewpoints. Journal of Experimental Child Psychology, 170, 1–29. 10.1016/j.jecp.2017.12.009 29407185

[c48] Negen J., Wen L., Thaler L., & Nardini M. (2018). Bayes-like integration of a new sensory skill with vision. Scientific Reports, 8(1), 16880. 10.1038/s41598-018-35046-7 30442895 PMC6237778

[c49] Newcombe N. S., Ratliff K. R., Shallcross W. L., & Twyman A. D. (2010). Young children’s use of features to reorient is more than just associative: Further evidence against a modular view of spatial processing. Developmental Science, 13(1), 213–220. 10.1111/j.1467-7687.2009.00877.x 20121877

[c50] Park S., Brady T. F., Greene M. R., & Oliva A. (2011). Disentangling scene content from spatial boundary: Complementary roles for the parahippocampal place area and lateral occipital complex in representing real-world scenes. The Journal of Neuroscience: The Official Journal of the Society for Neuroscience, 31(4), 1333–1340. 10.1523/JNEUROSCI.3885-10.2011 21273418 PMC6623596

[c51] Petrini K., Remark A., Smith L., & Nardini M. (2014). When vision is not an option: Children’s integration of auditory and haptic information is suboptimal. Developmental Science, 17(3), 376–387. 10.1111/desc.12127 24612244 PMC4240463

[c52] Pouget A., Beck J. M., Ma W. J., & Latham P. E. (2013). Probabilistic brains: Knowns and unknowns. Nature Neuroscience, 16(9), 1170–1178. 10.1038/nn.3495 23955561 PMC4487650

[c53] Rahnev D., & Denison R. N. (2018). Suboptimality in perceptual decision making. Behavioral and Brain Sciences, 41, 1–107. 10.1017/S0140525X18000936 PMC6110994 29485020

[c54] Ratliff K. R., & Newcombe N. S. (2008a). Is language necessary for human spatial reorientation? Reconsidering evidence from dual task paradigms. Cognitive Psychology, 56(2), 142–163. 10.1016/j.cogpsych.2007.06.002 17663986

[c55] Ratliff K. R., & Newcombe N. S. (2008b). Reorienting when cues conflict: Evidence for an adaptive-combination view. Psychological Science, 19(12), 1301–1307. 10.1111/j.1467-9280.2008.02239.x 19121141

[c56] Rohde M., van Dam L. C. J., & Ernst M. (2016). Statistically optimal multisensory cue integration: A practical tutorial. Multisensory Research, 29(4-5), 279–317. 10.1163/22134808-00002510 29384605

[c57] Rouder J. N., Speckman P. L., Sun D., Morey R. D., & Iverson G. (2009). Bayesian t tests for accepting and rejecting the null hypothesis. Psychonomic Bulletin & Review, 16(2), 225–237. 10.3758/PBR.16.2.225 19293088

[c58] Sato Y., & Kording K. P. (2014). How much to trust the senses: Likelihood learning. Journal of Vision, 14(13), 13. 10.1167/14.13.13 PMC4233767 25398975

[c59] Sjolund L. A., Kelly J. W., & McNamara T. P. (2018). Optimal combination of environmental cues and path integration during navigation. Memory & Cognition, 46(1), 89–99. 10.3758/s13421-017-0747-7 28828745

[c60] Smith A. D., Hood B. M., & Gilchrist I. D. (2010). Probabilistic cuing in large-scale environmental search. Journal of Experimental Psychology: Learning, Memory, and Cognition, 36(3), 605–618. 10.1037/a0018280 20438260

[c61] Stürzl W., Cheung A., Cheng K., & Zeil J. (2008). The information content of panoramic images I: The rotational errors and the similarity of views in rectangular experimental arenas. Journal of Experimental Psychology: Animal Behavior Processes, 34(1), 1–14. 10.1037/0097-7403.34.1.1 18248111

[c62] Tassinari H., Hudson T. E., & Landy M. S. (2006). Combining priors and noisy visual cues in a rapid pointing task. The Journal of Neuroscience: The Official Journal of the Society for Neuroscience, 26(40), 10154–10163. 10.1523/JNEUROSCI.2779-06.2006 17021171 PMC6674625

[c63] Twyman A. D., Holden M. P., & Newcombe N. S. (2018). First direct evidence of cue integration in reorientation: A new paradigm. Cognitive Science, 42(Suppl. 3), 923–936. 10.1111/cogs.12575 29178140

[c205] Vizard. (2018). Vizard 5 [Computer software]. https://worldviz.com

[c64] Wang L., Mou W., & Dixon P. (2018). Cue interaction between buildings and street configurations during reorientation in familiar and unfamiliar outdoor environments. Journal of Experimental Psychology: Learning, Memory, and Cognition, 44(4), 631–644. 10.1037/xlm0000478 29094988

[c65] Xu Y., Regier T., & Newcombe N. S. (2017). An adaptive cue combination model of human spatial reorientation. Cognition, 163, 56–66. 10.1016/j.cognition.2017.02.016 28285237

[c66] Zhao M., & Warren W. H. (2015). How you get there from here: Interaction of visual landmarks and path integration in human navigation. Psychological Science, 26(6), 915–924. 10.1177/0956797615574952 25944773

